# Genome sequence diversity of SARS-CoV-2 in Serbia: insights gained from a 3-year pandemic study

**DOI:** 10.3389/fmicb.2024.1332276

**Published:** 2024-02-27

**Authors:** Mirjana Novkovic, Bojana Banovic Djeri, Bojan Ristivojevic, Aleksandra Knezevic, Marko Jankovic, Vanja Tanasic, Verica Radojicic, Dusan Keckarevic, Dejan Vidanovic, Bojana Tesovic, Anita Skakic, Maja Tolinacki, Ivana Moric, Valentina Djordjevic

**Affiliations:** ^1^Center for Genome Sequencing and Bioinformatics, Institute of Molecular Genetics and Genetic Engineering, University of Belgrade, Belgrade, Serbia; ^2^Institute of Microbiology and Immunology, Department of Virology, Faculty of Medicine, University of Belgrade, Belgrade, Serbia; ^3^Center for Forensic and Applied Molecular Genetics, Faculty of Biology, University of Belgrade, Belgrade, Serbia; ^4^Veterinary Specialized Institute “Kraljevo”, Kraljevo, Serbia

**Keywords:** SARS-CoV-2, genome, next generation sequencing, Serbia, COVID-19, pandemic

## Abstract

The severe acute respiratory syndrome coronavirus 2 (SARS-CoV-2), responsible for the COVID-19 pandemic, has been evolving rapidly causing emergence of new variants and health uncertainties. Monitoring the evolution of the virus was of the utmost importance for public health interventions and the development of national and global mitigation strategies. Here, we report national data on the emergence of new variants, their distribution, and dynamics in a 3-year study conducted from March 2020 to the end of January 2023 in the Republic of Serbia. Nasopharyngeal and oropharyngeal swabs from 2,398 COVID-19-positive patients were collected and sequenced using three different next generation technologies: Oxford Nanopore, Ion Torrent, and DNBSeq. In the subset of 2,107 SARS-CoV-2 sequences which met the quality requirements, detection of mutations, assignment to SARS-CoV-2 lineages, and phylogenetic analysis were performed. During the 3-year period, we detected three variants of concern, namely, Alpha (5.6%), Delta (7.4%), and Omicron (70.3%) and one variant of interest—Omicron recombinant “Kraken” (XBB1.5) (<1%), whereas 16.8% of the samples belonged to other SARS-CoV-2 (sub)lineages. The detected SARS-CoV-2 (sub)lineages resulted in eight COVID-19 pandemic waves in Serbia, which correspond to the pandemic waves reported in Europe and the United States. Wave dynamics in Serbia showed the most resemblance with the profile of pandemic waves in southern Europe, consistent with the southeastern European location of Serbia. The samples were assigned to sixteen SARS-CoV-2 Nextstrain clades: 20A, 20B, 20C, 20D, 20E, 20G, 20I, 21J, 21K, 21L, 22A, 22B, 22C, 22D, 22E, and 22F and six different Omicron recombinants (XZ, XAZ, XAS, XBB, XBF, and XBK). The 10 most common mutations detected in the coding and untranslated regions of the SARS-CoV-2 genomes included four mutations affecting the spike protein (S:D614G, S:T478K, S:P681H, and S:S477N) and one mutation at each of the following positions: 5′-untranslated region (5’UTR:241); N protein (N:RG203KR); NSP3 protein (NSP3:F106F); NSP4 protein (NSP4:T492I); NSP6 protein (NSP6: S106/G107/F108 - triple deletion), and NSP12b protein (NSP12b:P314L). This national-level study is the most comprehensive in terms of sequencing and genomic surveillance of SARS-CoV-2 during the pandemic in Serbia, highlighting the importance of establishing and maintaining good national practice for monitoring SARS-CoV-2 and other viruses circulating worldwide.

## 1 Introduction

The severe acute respiratory syndrome coronavirus 2 (SARS-CoV-2), responsible for the COVID-19 pandemic, was first detected in December 2019 in Hubei province of China. The disease has spread rapidly, forcing the World Health Organization (WHO) to declare a pandemic on 11 March 2020 (World Health Organization, 2020a). By early September 2023, there were more than 754 million confirmed cases of COVID-19 worldwide, including 6.85 million deaths (4 September 2023, daily online worldwide COVID-19 data).^1^ According to the Institute of Public Health of the Republic of Serbia “Dr Milan Jovanovic Batut”,^2^ the first case of COVID-19 was confirmed on 6 March 2020, and since then 2.5 million cases and approximately 18,000 deaths have been reported in Serbia^3^ (see text footnote 1) resulting in a mortality rate of 0.72%.

SARS-CoV-2 is classified in the genus *Betacoronavirus*, which belongs to the family Coronaviridae and the order Nidovirales (Liu et al., 2020). It was hypothesized that SARS-CoV-2, like most of the previously identified coronaviruses infecting humans (SARS-CoV, MERS-CoV), originated in bats and then bridged to humans via an intermediate host, possibly Malayan pangolins (Goraichuk et al., 2021). Additionally, genetic studies revealed 96.2% identity of SARS-CoV-2 to coronavirus RaTG13 isolated from horseshoe bats (*Rhinolophus affinis*) from China (Goraichuk et al., 2021), which underscores the need for continuous surveillance of coronaviruses (CoVs) present in animal reservoirs to prevent future transmission to humans.

The genome of SARS-CoV-2 is a positive-sense single-stranded RNA that is ~29,900 nucleotides long (Liu et al., 2020). The genome contains 14 open reading frames (ORFs) and encodes 27 proteins. The first ORF, ORF1ab, comprises approximately two-thirds of the genome and encodes 16 non-structural proteins needed for the replication of SARS-CoV-2 (Chan et al., 2020). The remaining ORFs encode for accessory and structural proteins. There are four major structural proteins: spike (S), membrane (M), envelope (E), and nucleocapsid (N). The N, M, and E proteins are necessary for virion morphogenesis, assembly, and release. The S protein of SARS-CoV-2 is a major determinant of the pathogenesis of viral infection since it is responsible for binding to human angiotensin-converting enzyme 2 (ACE2) receptor (Chan et al., 2020).

During the 3 years of the COVID-19 pandemic, the genome of SARS-CoV-2 has undergone major changes, some of which affected viral replication, transmission, pathogenicity, and virulence. The emergence of lineages that pose an increased risk to global public health prompted WHO to classify some of them as variant of concern (VOC), variant of interest (VOI), and variant under monitoring (VUM) and prioritize global surveillance and research to adjust the COVID-19 response. So far, five different lineages have been designated as VOCs: Alpha, Beta, Gamma, Delta, and Omicron (World Health Organization, 2023a).

According to WHO (see text footnote 1) and online worldwide COVID-19 data,^4^ throughout the time of our study (up to the end of January 2023), the world has experienced eight major COVID-19 pandemic waves. Even though the number of SARS-CoV-2 infections remained high worldwide in 2022, there was evidence of a decrease in severe COVID-19 cases (Veneti et al., 2022; Ward et al., 2022). This was interpreted as the result of the development of population immunity through infections and/or vaccination, improved health management, and, most importantly, lower virulence of the latest circulating Omicron sublineages (Wu et al., 2023). All of these factors have led to a significant global decline in the weekly number of COVID-19-related deaths and hospitalizations, and WHO declared the end of the emergency phase of COVID-19 in May 2023 (World Health Organization, 2020b). However, WHO continues to coordinate the global response and track new cases as SARS-CoV-2 continues to evolve and circulate.

Surveillance of SARS-CoV-2 at the national level proved to be of the utmost importance for early detection and monitoring of the spread of the virus, as well as for identifying and tracking the emergence of new variants. Monitoring the evolution of the virus provides valuable insights into public health interventions and the development of targeted control strategies. This helps policymakers and healthcare systems prepare for a potential expansion in cases, allocate resources efficiently, and mitigate the impact of the pandemic.

The aim of this study was to analyze genome sequence diversity of SARS-CoV-2 during the COVID-19 pandemic on the territory of the Republic of Serbia. Here, we report the emergence of new variants and their distribution and dynamics in a 3-year long study conducted in Serbia from March 2020 to the end of January 2023. This national-level study is the most comprehensive one regarding sequencing and genome monitoring of SARS-CoV-2 during the pandemic in Serbia. Our study contributes to the retrospective understanding of the global spread of variants during the pandemic. Additionally, it stresses the importance of establishing and maintaining good national practice for viral surveillance of SARS-CoV-2 and future virus outbreaks.

## 2 Materials and methods

### 2.1 Surveillance system

COVID-19 surveillance system in the Republic of Serbia was gradually built over 3 years. The first step was to appoint COVID-19 sampling and testing centers which were formed within the public and community health centers and outpatient clinics throughout Serbia. Additionally, qRT-PCR testing was performed at the Institute of Virology Vaccines and Sera “Torlak,” Center for Laboratory Medicine Clinical Center of Vojvodina (CLMV), Institute of Biocide and Medical Ecology, The Directorate for National Reference Laboratories, Scientific Institute for Veterinary Science “Novi Sad,” and other local veterinary science institutes in Serbia. Furthermore, in April 2020 and July 2020, two National Laboratories for Molecular Detection of Infectious Agents “Huo-Yan” [Clinical Center of Serbia in Belgrade (HYB) and Clinical Center of Nis (HYN)] were established and started with massive qRT-PCR testing of SARS-CoV-2 samples. All the data regarding COVID-19 testing were deposited to a newly established centralized database for the Republic of Serbia, in which the testing laboratories uploaded results based on the sample barcode with no access to patient data. Access to patient data was permitted only to medical doctors.

In parallel, local scientific institutes with Next Generation Sequencing (NGS) capacities started with SARS-CoV-2 sample sequencing. At the very beginning of pandemic, sequencing was performed for research purpose and was based on collaboration between testing laboratories and scientific institutes. Accordingly, the first 48 samples were sequenced at the Veterinary Specialized Institute “Kraljevo” in real time. After that, two other laboratories at the Institute of Microbiology and Immunology, Department of Virology (Faculty of Medicine, University of Belgrade), and Center for Forensic and Applied Molecular Genetics (Faculty of Biology, University of Belgrade) were also engaged in SARS-CoV-2 sequencing. However, during the first 2 years of pandemic, sequencing was mostly performed with a delay in comparison to the date of sample collection.

At the end of 2021, the Government of the Republic of Serbia supported establishing of the Center for Genome Sequencing and Bioinformatics (CGSB) at the Institute of Molecular Genetics and Genetic Engineering University of Belgrade which led to the development of a stable system for positive sample collection and their regular monthly sequencing in real time from April 2022 onward. Samples were collected from three major regional centers for SARS-CoV-2 testing (CLMV, HYB, and HYN) and covered the majority of the regions in Serbia. Any newly identified variants were reported to the Crisis Response Team of the Republic of Serbia.

### 2.2 Sample collection

Clinical specimen samples used in this study were collected as nasopharyngeal and oropharyngeal swabs which were taken by medical professionals in the public health centers from the patients with COVID-19-like symptoms between 9 March 2020 and 31 January 2023 on the territory of the Republic of Serbia. These specimen samples were used for detecting the presence of SARS-CoV-2 using qRT-PCR. Samples with Ct values <25 were selected for sequencing.

All specimens used in this study were previously anonymized containing only the information regarding the city of residence and, in some cases, age and gender of patient, all provided by the public health centers where specimens were collected.

### 2.3 SARS-CoV-2 NGS analysis

Genome sequencing was performed in four different laboratories using three different NGS technologies: Oxford Nanopore Technologies (ONT), Ion Torrent Technology (ITT), and DNBSeq Technology (DNBST). The ONT platform was used for SARS-CoV-2 sequencing in the Veterinary Specialized Institute “Kraljevo” and at the Institute of Microbiology and Immunology, Department of Virology, Faculty of Medicine, University of Belgrade (Vidanović et al., 2021). At the Center for Forensic and Applied Molecular Genetics (Faculty of Biology, University of Belgrade), sequencing was carried out using ITT platform, while at the Center for Genome Sequencing and Bioinformatics at the Institute of Molecular Genetics and Genetic Engineering, University of Belgrade, DNBS platform was used. The details of all three sequencing protocols are presented in Supplementary material.

Analysis of NGS data was performed in accordance with the recommendation for each platform: ARTIC nCoV bioinformatics standard operating procedure v.1.1.0 (Loman et al., 2020) using the settings as in Vidanović et al. (2021), Torrent Suite software v. 5.10.1 with SARS-CoV-2 Research Plug-in Package (Coverage Analysis, Variant Caller, COVID19AnnotateSnpEff, IRMAreport and AssemblerTrinity) based on the alignment to the Wuhan-Hu-1 NCBI Genome (MN908947.3*), and “SARS-CoV-2 Genome Assembly: Health Analysis in One-Step Machine V2.0” (GA HALOS V2.0) system (BGI Tech, Shenzhen, China) with SARS-CoV-2 (NC_045512.2*) built-in pipeline for alignment and reference-based assembly (including fastp trimming, FLASH merging paired-end reads, BWA alignment, read manipulation, freebayes variant calling, SnpEff annotation, and SPAdes assembly) were used for ONT, ITT and DNBST read analysis, respectively. Notably, NC_045512.2 and MN908947.3 have identical nucleotide sequences, despite different accession numbers. The sequence analysis was performed by the researchers who carried out sequencing.

### 2.4 Sanger sequencing

Sanger sequencing with appropriate primers from the ARTIC Network was used for long gaps (>100 bp) when needed to confirm certain single nucleotide polymorphisms (SNPs) and frameshift deletions. Sequencing reactions were performed using the BigDye Terminator Kit v3.1 (Applied Biosystems) according to the manufacturer’s instructions and run on the Genetic Analyzer 3130 (Applied Biosystems).

### 2.5 Data deposition

All 2,398 SARS-CoV-2 genome sequences from the Republic of Serbia have been deposited in the GISAID^5^ EpiCoV database (the Global Initiative on Sharing all Individual Data) with specified location as Europe/Serbia/City Name. The data were deposited by the researchers who performed sequencing (submitter indicated in GISAID).

### 2.6 Mutation analysis

In the subset of 2,107 SARS-CoV-2 sequences in Serbia variant detection, analysis of the overall mutations per sample, the most mutated samples, the most frequent mutation per class, and the most frequent mutations in coding and untranslated regions were performed using the Coronapp^6^ tool (Mercatelli et al., 2020).

### 2.7 Phylogenetic analysis

The clade and lineage assignments for each of 2,398 SARS-CoV-2 genome sequences were uniformly performed using Nextclade web version 2.14.1 (Aksamentov et al., 2021). For the phylogenetic analysis and the mutation analysis, the subset of 2,107 SARS-CoV-2 sequences available as the EPI_SET_230901st was used.^7^ The subset included sequences with coverage greater than 98.0% and less than 1% of unidentified nucleotides (N’s), according to the requirements for sequences designated as those with high coverage (APHL, 2021). The phylogenetic analysis was performed using the Augur bioinformatics toolkit v.22.0.3, while for phylogenetic data visualization, the Auspice interactive tool v.2.47.0 was used (Hadfield et al., 2018; Huddleston et al., 2021). The tree was rooted with sequence NC_045512.2 (Wuhan-Hu-1) on 31 December 2019, which was sufficiently timely distant from analyzed samples and also used as the reference sequence by Nextstrain.

## 3 Results

In this study, we report the results of genomic surveillance of the SARS-CoV-2 virus on the territory of the Republic of Serbia during the COVID-19 pandemic from March 2020 to January 2023. During this 3-year long study, we have sequenced 2,398 SARS-CoV-2 samples collected from different regions of the Republic of Serbia, including Province of Vojvodina, Central, Eastern, Western, Southern Serbia, Province of Kosovo and Metohija, and Belgrade municipality. Out of them, 291 samples were of insufficient quality, and although their clade and lineage could be assigned in all but two cases (Supplementary Table 1), they were excluded from the study in order to not to interfere with the overall mutational and phylogenetic analyses. Therefore, we analyzed the subset of 2,107 sequences ranging from 29,475 to 29,870 nucleotides in length, all fulfilling conditions of ≥98.0% coverage and ≤ 1% of ambiguous sites.

The average number of samples sequenced per month was approximately 62, with a minimum of 10 samples in March 2022 and maximum of 289 samples in May 2022. The lower number of samples per month was a consequence of generally low sampling rates (e.g., September 2020), sample degradation due to the inappropriate storage (e.g., June 2021), or the use of a viral transport medium that was not suitable for the downstream sequencing application (September 2021).

**Figure 1:**
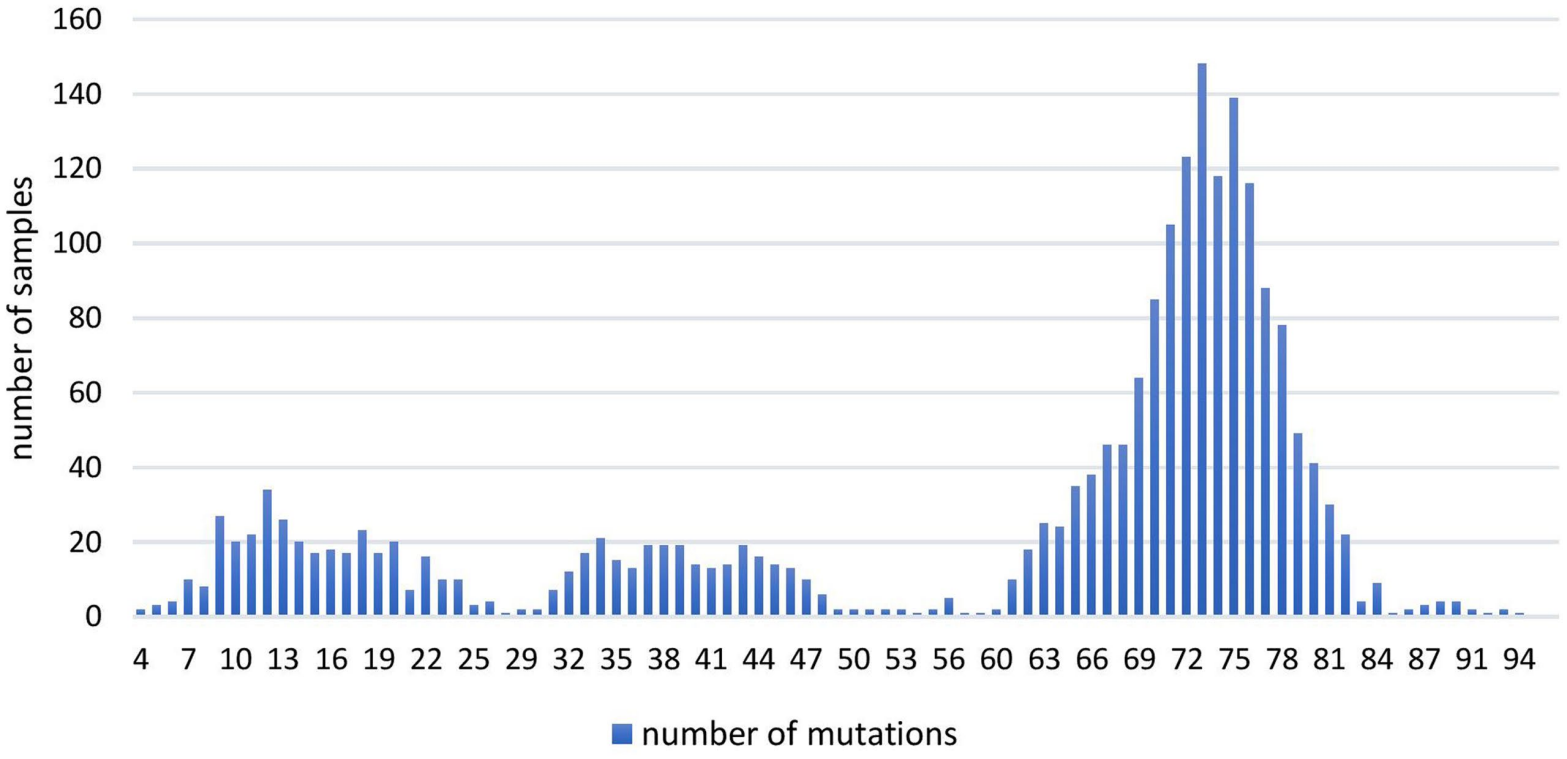
The number of overall mutations per sample in 2,107 SARS-CoV-2 sequences obtained in the period March 2020–January 2023 on the territory of the Republic of Serbia.

The analysis of the total number of genomic mutations per sample (Figure 1) showed variations from 4 to 94. All the sequences containing 58 or more genomic mutations belonged to different Omicron (sub) lineage.

Ten most mutated samples belonged to different sublineages of the Omicron variant, four of which were detected during 2022, while the remaining six were detected in January 2023 on the territory of the Republic of Serbia (Table 1).

**Table 1:** Ten most mutated samples in a subset of 2,107 SARS-CoV-2 sequences analyzed in the period March 2020–January 2023 on the territory of the Republic of Serbia (all ten Omicron sublineages).

Number of mutations per genome	Place of sampling	Date of sampling	EPI number	Omicron variant sublineage
94	Belgrade municipality	May 2022	EPI_ISL_13311315	BA.2.72
93	Southern Serbia	January 2023	EPI_ISL_17250971	XBB.2
	Southern Serbia	January 2023	EPI_ISL_17250969	BN.1.3.1
92	Southern Serbia	January 2023	EPI_ISL_17251030	XBB.1.5
91	Province of Vojvodina	October 2022	EPI_ISL_16316940	BN.1.3.1
	Belgrade municipality	November 2022	EPI_ISL_16317018	BL.1
90	Belgrade municipality	November 2022	EPI_ISL_16316948	XBB.1
	Southern Serbia	January 2023	EPI_ISL_17251024	BN.1
	Central Serbia	January 2023	EPI_ISL_17251028	BA.5.2
	Central Serbia	January 2023	EPI_ISL_17250973	XBB.2

**Figure 2:**
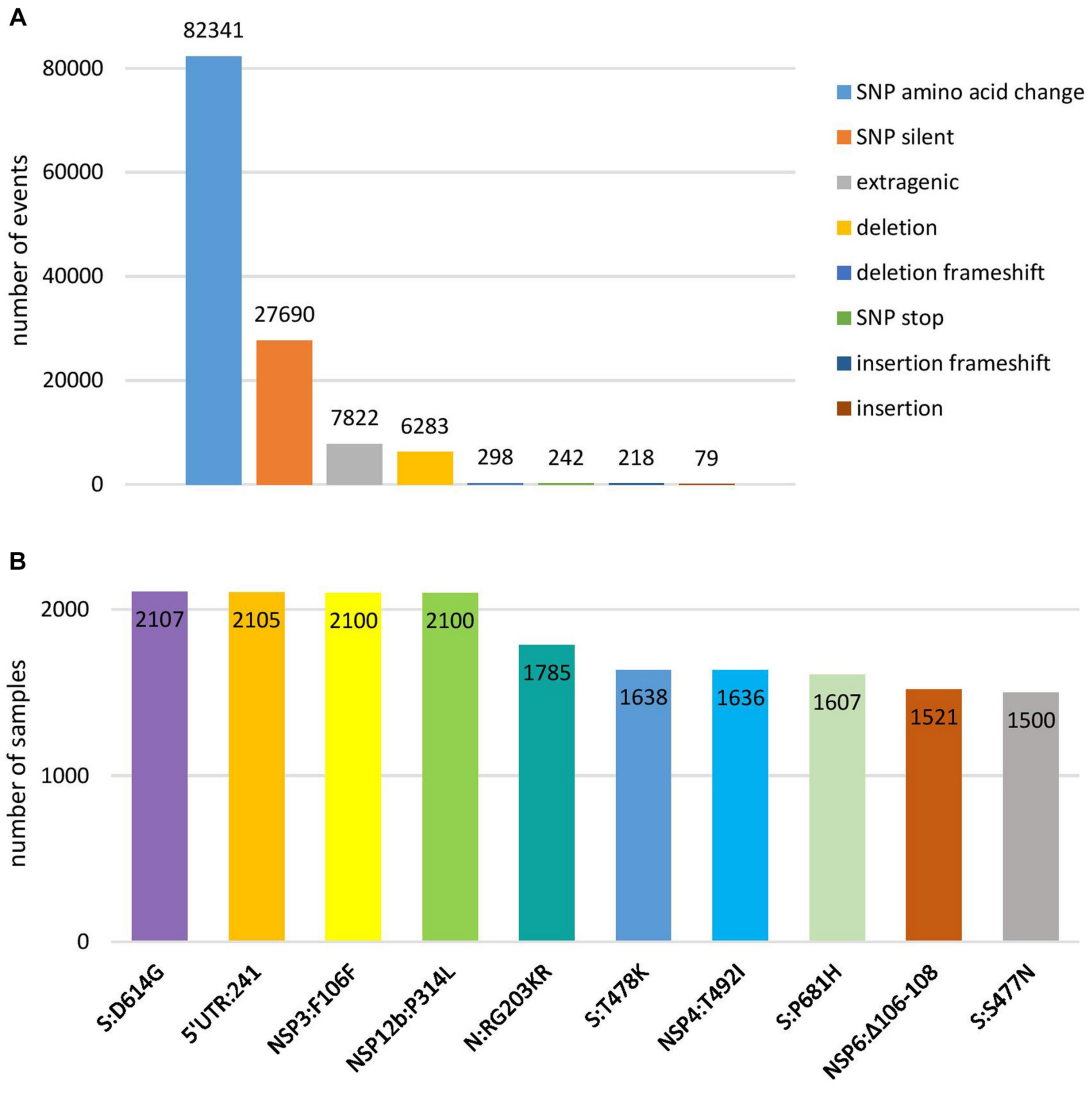
Overview of the most frequent types of mutations at nucleotide level **(A)** and ten most frequent mutations in coding and untranslated regions **(B)** detected in 2,107 SARS-CoV-2 sequences on the territory of the Republic of Serbia in the period March 2020–January 2023.

Genomic variant analysis revealed nearly 125,000 mutations in the 2,107 sequences, of which 38.9% occurred in the spike protein-coding gene. The most frequent type of intragenic mutations (Figure 2A) were single nucleotide polymorphisms (SNPs) (88%), followed by intragenic deletions (5%). Among detected SNPs, 65.9% caused amino acid substitutions, 22.1% were silent, and 6.2% were extragenic, while 0.2% caused a premature stop codon. The ten most frequent mutations detected in coding and untranslated regions (Figure 2B) included four mutations that affected the spike protein (S:D614G, S:T478K, S:P681H, and S:S477N) and one mutation at each of the following positions: 5′ untranslated region (5’UTR:241); N protein (N:RG203KR); NSP3 protein (NSP3:F106F); NSP4 protein (NSP4:T492I); NSP6 protein (NSP6: S106/G107/F108 - triple deletion), and NSP12b protein (NSP12b:P314L). The detailed findings regarding these mutations are presented in Table 2.

**Table 2:** Overview of the ten most frequent mutations in a subset of 2,107 SARS-CoV-2 sequences analyzed in the period March 2020–January 2023 on the territory of the Republic of Serbia.

	Position of mutation	Percentage^$^ (total number)	Exact change	Timespan of appearance in Serbia	Functional effects
1	S:614	100 (2107)	D614G	March 2020^*^	Increases viral replication in human lung epithelial cells, boosts viral loads in the COVID-19 patients’ upper respiratory tract (Korber et al., 2020; Li et al., 2022)
2	5’UTR	99.9 (2015)	C241T^#^	March 2020^*^	Most likely impacts viral RNA folding, packaging and titres (Mishra et al., 2020)
3	NSP3:106	99.7 (2100)	F106F	March 2020^*^	Possibly changes the codon usage and translation efficiency of NSP3, favouring the viral infection (Majumdar and Niyogi, 2021)
4	NSP12b:314	99.7 (2100)	P314L	March 2020^*^	Increases viral transmissibility in cooperation with D614G (Wang et al., 2021)
		<0.1 (5)	P314F	July 2022 – November 2022	Potentially impacts viral replication due to a change of fitness (Proust et al., 2023)
5	N:203/204	84.7 (1785)	R203K & G204R	March 2020^*^	Increases the binding of virus to cells (Raheja et al., 2022)
		8.0 (168)	R203M only	April 2020 – February 2022	Leads to increased viral mRNA packaging and delivery (Syed et al., 2021)
		<0.1 (44)	G204R only	August 2021 – January 2023	Information not available
		<0.1 (4)	R203K & G204P	May 2021, August 2022	
		<0.1 (1)	R203K & G204L	March 2021	
6	S:478	77.7 (1638)	T478K	End of June 2021^*^	Probably affects viral infectivity and pathogenesis by changing spike gene’s receptor binding motif (Jhun et al., 2021)
7	NSP4:492	77.6 (1636)	T492I	End of June 2021^*^	Boosts viral transmissibility and infectivity by increasing the replication capacity and ability to evade host immune responses (Lin et al., 2023)
8	S:681	76.3 (1607)	P681H	December 2020^*^	Initially considered to enhance resistance to interferon-β through S cleavage (Lista et al., 2022), which was later refuted, suggesting other mutations associated with it may contribute to viral replication/transmission advantages (Lubinski et al., 2022)
9	NSP6:Δ3 amino acids	72.2 (1521)	Δ(S106/G107/F108)	December 2020^*^	Antagonizes type I interferon response and provides fitness advantage (Hossain et al., 2022; Bills et al., 2023)
		4.0 (85)	Δ(L105/S106/G107)	December 2021 – June 2022	Facilitates autophagy by favouring interaction with membrane (Hossain et al., 2022)
10	S:477	71.2 (1500)	S477N	September 2020^*^	Strengthens viral binding to the human ACE2 receptor (Singh et al., 2021)

^$^Percentage calculated based on the total number of 2,107 samples; ^*^Mutation present in all samples until the end of the study period; ^#^Nucleotide change.

**Figure 3:**
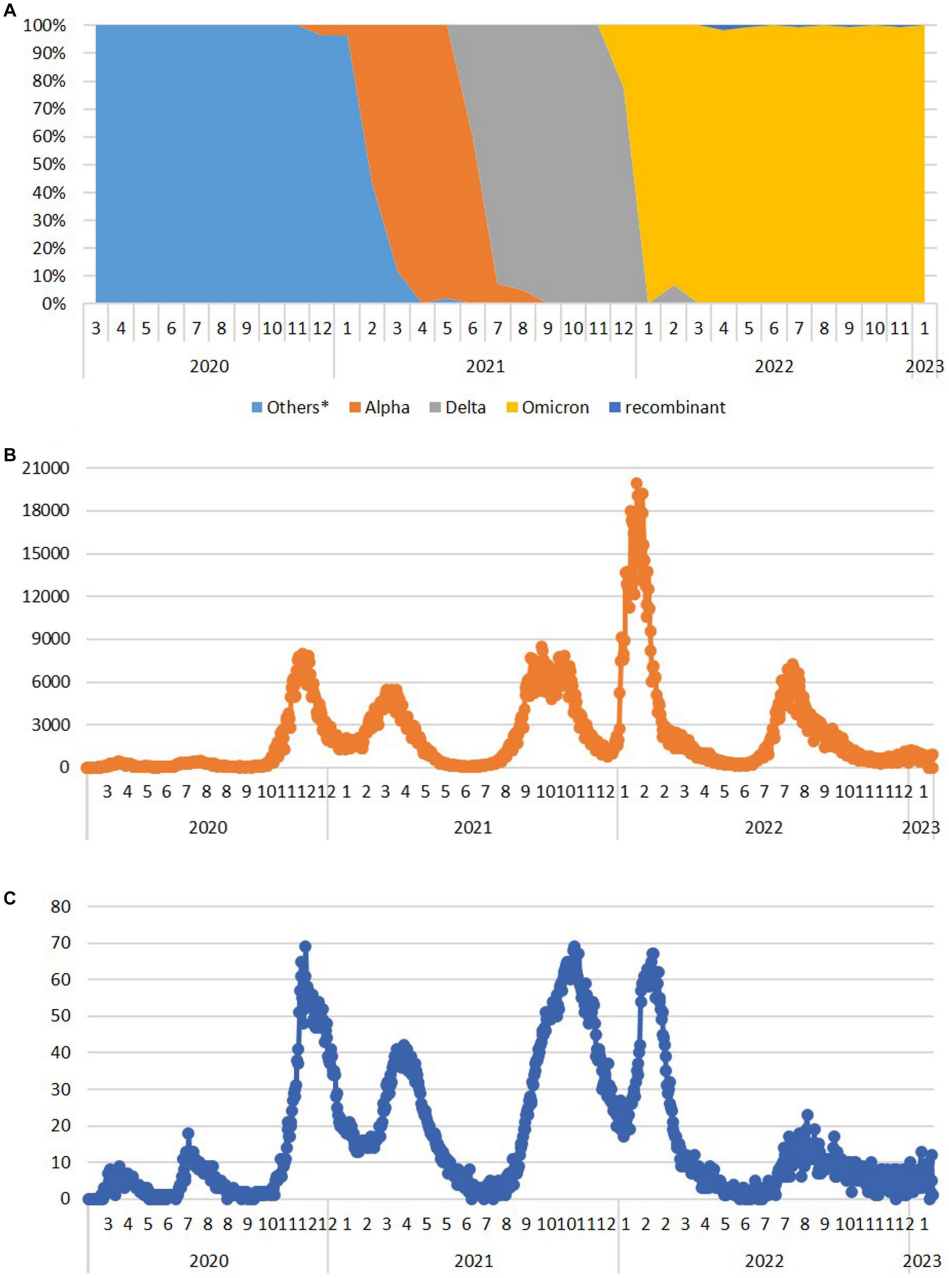
The dynamics of the appearance and dominance of SARS-CoV-2 variants formerly designated as variants of concern during the 3-year long surveillance in the Republic of Serbia during the period March 2020–January 2023 **(A)** and the number of COVID-19 new daily cases **(B)** and deaths **(C)** available at http://www.worldometers.info/coronavirus/country/serbia (Others* - sequences belong to the Nextstrain clades 20A, 20B, 20C, 20D, 20E, and 20G, which have not been assigned by WHO).

Several of the aforementioned mutations were associated with the emergence of lineages designated as VOCs. Here, we report the timeline of detection and dominance of these VOCs in Serbia. The Alpha variant (B.1.1.7) was detected for the first time in Serbia on 13 December 2020, remaining present until August 2021. It was dominant from February 2021 to June 2021, reaching the maximum frequency of 100% in analyzed sequences in April 2021 (Figure 3A). This variant contributed 5.6% (119/2,107) to total SARS-CoV-2 (sub) lineage detected in Serbia during the study period.

The first case of the Delta variant (AY.122) was detected in Serbia on 26 June 2021, and this VOC remained present until the end of December 2021. Immediately after its appearance, it accounted for 41.7% of all variants detected in June 2021. In July 2021, it became the dominant variant representing 92.9% of all detected variants, finally reaching 100% during September, October, and November 2021, until Omicron appeared in December 2021 (Figure 3A). We detected 24 different Delta sublineages among which AY.122 (32.5%), AY.43 (26.1%), AY.46.6 (17.2%), and B.1.617.2 (4.5%) were the most abundant ones. The Delta variant contributed 7.4% (157/2,107) to total SARS-CoV-2 sequences in this study.

The first Omicron case (BA.1.1) was detected in Serbia on 12 December 2021, and this VOC remained present until the end of the study. Omicron overtook the dominance very fast, reaching a frequency of 100% in Serbia already in January 2022 (Figure 3A). We detected 113 sublineages of which BA.2.* (35.2%), BA.5.* (42.8%), BA.1.* (5.7%), BF.14 (3.6%), and BE.1.1 (2.9%) were the most abundant ones. The Omicron variant contributed 70.3% (1,481/2107) to total SARS-CoV-2 sequences in this study.

Six Omicron recombinants were detected in Serbia during this study, but not all of them met the criteria to be included in the subset of analyzed 2,107 sequences. In addition to 5 XZ detected from April 2022 and 3 XAZ sequences detected from July 2022, only 2 out of 25 XBB.1.5 (“Kraken”) sequences detected in January 2023 met the quality requirements to be included in this study, while 3 XAS recombinants detected in September 2022 and 1 XBF and 1 XBK recombinant detected in January 2023 were not included in the subset of 2,107 sequences.

The remaining 16.8% of SARS-CoV-2 (sub) lineage detected in Serbia belonged to other (sub) lineage which were not classified as VOC, VOI, or VUM.

We did not detect any Beta (B.1.315 + B.1.315.2 + B.1.315.3), Gamma (P.1 + P.1*), Lambda (C.37 + C.37.1), Epsilon (B.1.427, B.1.429), Eta (B.1.525), Theta (P3), Iota (B.1.526), Kappa (B.1.617.1), Lambda (C.37), Zeta (P.2), and Mu (B.1.621, B.1.621.1) variants in Serbia.

Analyzing the number of COVID-19 new daily cases (ndc) and deaths (ndd) from March 2020 until the end of January 2023 in Serbia (Serbia COVID, 2020), there were eight COVID-19 waves (Figure 3). The first two COVID-19 waves in Serbia were detected in April 2020 and in July 2020 (with peaks of 445 and 467 ndc on 16 April 2020 and 26 July 2020, respectively, and a peak of 18 ndd on 10 July 2020), followed by two greater waves from second half of October 2020 to January 2021 (third wave with a peak of nearly 8,000 ndc on 2 December 2020 and 69 ndd on 4 December 2020) and from January 2021 to June 2021 (fourth wave with a peak of nearly 5,500 ndc on 23 March 2021 and two peaks of 41 ndd on 5 January 2021 and 29 March 2021). After the second and fourth wave, ndc number decreased below 500 and ndd below 10, while between the third and fourth waves, ndc remained at 1,300 and above and ndd between 13 and 41. The fifth wave lasted from the second half of July 2021 to December 2021 (with a peak of nearly 8,500 ndc on 28 September 2021 and of 69 ndd on 7 November 2021), while the sixth and biggest wave lasted from January 2022 to April 2022 (with a peak of nearly 20,000 ndc on 25 January 2022 and 67 ndd on 14 February 2022) and between these two waves, ndc number remained at 1000 and above and ndd to 20–35. In May 2022 and June 2022, the number of ndc decreased to 200–600 and ndd to 0–8, only to rise again from the end of June 2022 to November 2022 (with a peak of 7,265 ndc on 9 August 2022 and of 23 ndd on 26 August 2022) representing the seventh wave. At the end of 2022 and the beginning of 2023, new rise in ndc number was recorded, marking a considerably smaller eighth wave (the last one recorded in Serbia during this study), lasting from the end of December 2022 to the nearly mid-January 2023 (with a peak of 1,235 ndc on 4 January 2023 and 13 ndd on 16 January 2023).

**Figure 4:**
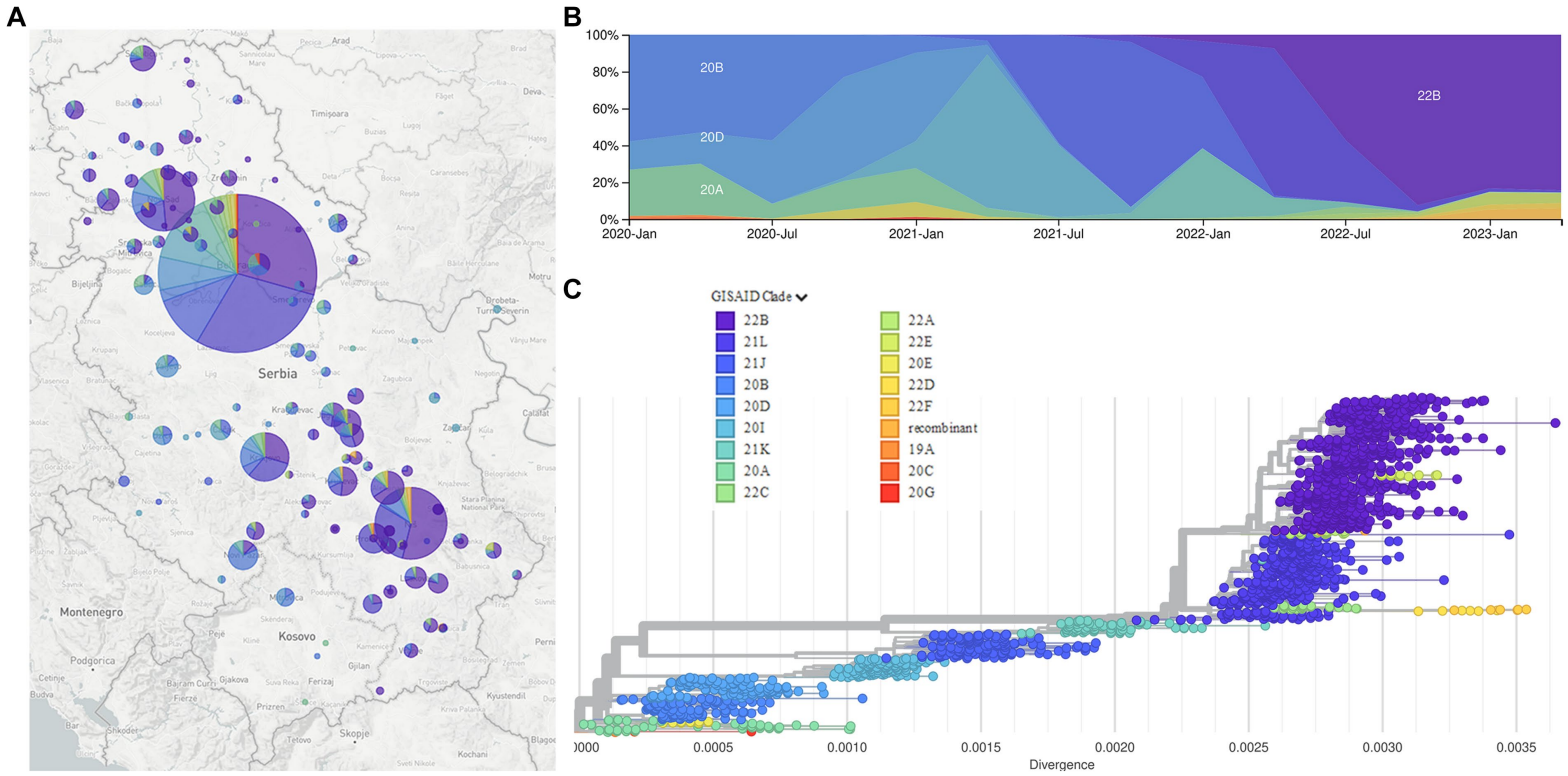
The Nextstrain clades of 2,107 SARS-CoV-2 sequences from the Republic of Serbia in the period March 2020–January 2023: Geographical distribution **(A)**; time distribution **(B)**; phylogenetic tree obtained using Auspice and Augur tools **(C)**.

Phylogenetic analysis of 2,107 SARS-CoV-2 sequences from Serbia shows the dynamic of SARS-CoV-2 genome changes on the territory of the Republic of Serbia starting from the first collected sample on 9 March 2020, which belonged to B.1.1.70 sublineage, until the last collected sample in this study on 26 January 2023, which belonged to BA.5.1.12 sublineage (Figure 4, interactive phylogenetic map available at https://nextstrain.org/community/novkovicm/SARS-CoV-2). The phylogenetic tree (Figure 4C) shows six larger clusters around clades 20A, 20B, 20I, 21J, 21K, and 21L. The first cluster included clades 20A, 20C, 20E, and 20G, the second cluster involved clades 20B and 20D, the third cluster involved clade 20I (Alpha variant), and the fourth cluster involved clade 21J (Delta variant). The fifth cluster included clade 21K (Omicron variant), while the sixth and largest cluster included clades 21L, 22A, 22B, 22C, 22D, 22E, and 22F and recombinants (Omicron variant). Clusters were arranged in that particular order looking from the bottom of the tree, which was rooted with reference sequence Wuhan-Hu-1 (NC_045512.2).

## 4 Discussion

In the battle against the COVID-19 pandemic, monitoring SARS-CoV-2 variants at the national level played an important role in understanding the dynamics of virus spread and adjusting public health measures accordingly. During the national surveillance in Serbia from March 2020 to January 2023, which included 2,107 SARS-CoV-2 sequences, the types and positions of the overall mutations and assigned variants were consistent with the global reports for this period (Urhan and Abeel, 2021; Chen et al., 2022).

Taking into account the total number of identified mutations at the nucleotide level, more than one-third (38%) occurred in the spike protein-encoding gene, which is further well correlated with the finding that among the ten most frequent mutations detected in coding and untranslated regions, four were found in the spike protein, namely, D614G, T478K, P681H, and S477N. Additionally, local samples showed a considerable number of unique mutations, yet the impact of these mutations on the fitness of the specific (sub) lineage is currently unknown. The presence of these unique, also called private mutations, which are not observed in the nearest neighbor in the Nextclade’s phylogenetic tree and are considered specific to individual samples, is a typical characteristic of SARS-CoV-2 and similar viruses.

Among the ten most frequent mutations identified in coding and untranslated regions, four were detected together in SARS-CoV-2 sequences in Serbia: 5’UTR:241, S:D614G, NSP12b:P314L, and NSP3:F106F, which is not surprising since they always co-occur in the same genomes, defining the major GISAID clade G and its derivative clades (Mercatelli and Giorgi, 2020). The fifth most frequent mutation of two amino acid changes at adjacent positions 203 and 204 in the N protein-coding gene (R203K and G204R/P/L) co-occurred with the four previously mentioned ones, representing GISAID GR clade (Majumdar and Niyogi, 2021). Sixth and seventh most frequent mutations (S:T478K and NSP4:T492I) were jointly detected in samples in Serbia as were eight and ninth mutations (S:P681H and NSP6: S106/G107/F108 - triple deletion).

Functionally, each of these mutations has brought significant advantage to viral fitness in different ways through improving SARS-CoV-2 replication, packaging, transmission, interaction with human receptors, and evasion of host immune system (Table 2) (Rambaut et al., 2020; Jhun et al., 2021; Volz et al., 2021; Hossain et al., 2022; Lista et al., 2022; Lubinski et al., 2022), supporting their high frequency detected in the local population. However, at the positions of the ten most frequent mutations, some alternative changes with minor frequencies (0.002–7.97%) were also detected (Table 2), suggesting that these most likely did not increase the viral fitness to the significant extent to be promoted further. Additionally, according to Nextstrain, several reversions to root were detected with minor frequencies (≤0.02%) as expected.

Regarding the detection and timeline of globally identified variants formerly designated as VOC and VOI by WHO (World Health Organization, 2023a), we have noticed a slight delay in the emergence of some (sub) lineage in Serbia, potentially attributed to a lower travel rate during 2020 and 2021 and also due to a decreased frequency of sequencing in certain months.

The Alpha variant (B.1.1.7 + Q.*), reported as the United Kingdom variant, was detected in Serbia approximately 3 months after its first official report in September 2020 (Public Health England, 2020; Duong, 2021). Its appearance in Serbia was correlated with the second half of the third local COVID-19 wave, while the fourth wave corresponded to the dominance of the Alpha variant in Serbia.

The Delta variant (B.1.617.2 + AY.*), reported to emerge in India in September 2020, was detected in Serbia approximately 9 months later (McCrone et al., 2021). Globally, the Delta variant became dominant in June 2021, while in Serbia, it became dominant in July 2021, causing the fifth local COVID-19 wave.

The Omicron variant (B.1.1.529.* + BA.*), reported to appear in South Africa and Botswana, was detected in Serbia only a month away from its first report (Ren et al., 2022). Our data suggest that this variant may have been introduced in Serbia by a traveler who came from Botswana in December 2021 (EPI_ISL_8748599). The overall high percentage of Omicron sublineages in this study, aside from its longest period of dominance, can be attributed also to the much higher number of samples sequenced at that period of the surveillance. Appearance of Omicron correlates with the sixth and the highest infection peak (nearly 20,000 ndc) during the pandemic in Serbia. In addition to Omicron appearance, the highest peak correlates with the period of New Year holidays, which has not been accompanied with additional measures for mitigating the pandemic (The Government of Republic of Serbia, 2021; The Government of Republic of Serbia, 2022). Moreover, the remaining presence of the Omicron variant in Serbia was responsible for the seventh and eighth pandemic waves as the last ones recorded during this study.

The XBB1.5 (“Kraken”), the current VOI, was reported to appear at the end of October 2022 in the United States and was detected in Serbia approximately 2 months later (World Health Organization, 2023b). This recombinant Omicron sublineage was at low frequency in the European Union at the end of December 2022 while becoming dominant in the United States at the end of January 2023 (Parums, 2023). Since we detected XBB1.5 at the end of the study period and the majority of its sequences were excluded from the study due to quality issues, it was difficult to discuss the “Kraken” influence and spread in Serbia as well as for other recombinant lineages XZ, XAZ, XAS, XBF and XBK, which were also detected in a very low number.

We did not detect any Beta, Gamma, Epsilon, Zeta, Eta, Theta, Iota, Lambda, Kappa, or Mu variants in Serbia, which may be explained by their geographical distribution, short period of existence, strict epidemiological measures in Serbia, and relatively low frequency of sequencing at the time of their circulation (World Health Organization, 2023c).

With respect to the number of recorded COVID-19 infections in Serbia, the low number of new cases per day reported in March 2020 could be explained by a state of emergency with a lockdown, which was effective from 15 March 2020 to 6 May 2020 (The Government of Republic of Serbia, 2020; International Monetary Fund, 2021), while relaxation of public health social and physical distancing measures for COVID-19 introduced from April 2020 was followed by the first COVID-19 wave in Serbia. Further removal of restrictions on the number of people allowed in outdoor public gatherings/events from 5 June 2020 (International Monetary Fund, 2021) was in correlation with the second COVID-19 wave in Serbia. Both waves correspond to the circulation of the SARS-CoV-2 lineages and sublineages, which have not been assigned by WHO. It is notable that the three highest ndd peaks in Serbia with ~70 deaths per day (Figure 3B - peaks III, V, and VI) correlate with the emergence of Alpha, Delta, and Omicron variants, respectively. The third ndc and ndd peaks correlate with the end of 2020, the period just before vaccination has started in Serbia. Furthermore, for peaks V and VI, corresponding to Delta and Omicron variants (respectively), a significant difference in the number of new daily cases (9,000 vs. 20,000), but comparable to death counts (~70 for both), was observed. These data clearly present the difference between COVID-19 severity and outcome, depending on the SARS-CoV-2 variant which are causing it (Veneti et al., 2022; Ward et al., 2022).

All eight detected waves recorded in Serbia correspond to the ones reported in Europe and the United States, as these two were roughly synchronized during the pandemic (Schellekens, 2023). The dynamics of pandemic waves in Serbia showed the most resemblance with the Southern Europe waves profile, which is understandable having in mind the southeast European position of Serbia.

Phylogenetic analysis of 2,107 SARS-CoV-2 sequences from Serbia during a 3-year long surveillance period showed that clades 20A, 20C, 20E, and 20G were grouped together in one cluster, while clades 20B and 20D were grouped in another cluster, close to the former one, as observed in the independent phylogenetic tree presented in other reports (Wu et al., 2021). The remaining four clusters included clades 20I (Alpha variant) and 21 J (Delta variant) as two smaller separate clusters, clade 21 K as another smaller cluster, and clades 21 L, 22A, 22B, 22C, 22D, 22E, 22F and recombinants as one larger cluster. This larger cluster was closely related to clade 21 K, which is understandable since both clusters represent different Omicron variants, with the larger one containing additional mutations and recombinations in comparison to the smaller one.

This study has some limitations. The number of samples sequenced per month varied greatly—from 10 to 289 samples; therefore, the frequencies of certain variants in the total number of sequences may not be as meaningful as their frequencies in the period of their circulation/presence. Low sampling/sequencing rates at certain months may cause some (sub) lineage to go undetected. Even though this study is a retrospective one and does not depend on the real-time tracking of SARS-CoV-2, from the perspective of the importance of real-time surveillance, we missed (sub) lineage tracking in the immediate time of their appearance during the first 2 years of the pandemic due to the lack of systematic sample collection and insufficient sequencing capacities. Only the first 48 samples were sequenced in the real time at the Veterinary Specialized Institute “Kraljevo” (Vidanović et al., 2021), while some others from this period were sequenced with the delay of up to a year. However, from April 2022 onward, we established a stable system for positive sample collection and regular monthly sequencing at the Center for Genome Sequencing and Bioinformatics at the Institute of Molecular Genetics and Genetic Engineering, so the number of samples highly increased in comparison to the earlier period of the study.

In this way, a considerable progress has been made in establishing and strengthening national systems to detect VOIs, VOCs, and VUMs, and it is important to maintain these systems operating and continue to share data in real time, since the circulation of SARS-CoV-2 continues globally (World Health Organization, 2023d). In conclusion, viral genome surveillance is of paramount importance in fighting pandemics such as COVID-19 because by utilizing these surveillance data, public health authorities can implement effective strategies to control the spread of the virus, mitigating the impact of the pandemic and keeping the vigilance at high level both nationally and globally.

## Data availability statement

The original contributions presented in the study are publicly available. This data can be found at: https://gisaid.org/, doi:10.55876/gis8.230901st.

## Ethics statement

The study was approved by ethics committee of the Institute of Molecular Genetics and Genetic Engineering, University of Belgrade (O-EO-058/2023). All specimens used in this study came from the secondary sources and were previously anonymized.

## Author contributions

MN: Conceptualization, Data curation, Formal analysis, Investigation, Supervision, Validation, Visualization, Writing – original draft, Writing – review & editing. BB: Conceptualization, Data curation, Formal analysis, Validation, Writing – original draft, Writing – review & editing. BR: Data curation, Investigation, Validation, Writing – review & editing. AK: Data curation, Funding acquisition, Investigation, Resources, Supervision, Writing – review & editing. MJ: Data curation, Investigation, Writing – review & editing. VT: Data curation, Investigation, Writing – review & editing. VR: Investigation, Writing – review & editing. DK: Funding acquisition, Investigation, Supervision, Writing – review & editing. DV: Data curation, Investigation, Project administration, Resources, Supervision, Writing – review & editing. BT: Data curation, Investigation, Writing – review & editing. AS: Investigation, Writing – review & editing. MT: Data curation, Investigation, Supervision, Writing – review & editing. IM: Data curation, Investigation, Writing – review & editing. VD: Funding acquisition, Project administration, Resources, Supervision, Writing – review & editing.

## Footnotes

^1^ https://covid19.who.int/

^2^ https://covid19.rs/the-first-case-of-covid-19-in-serbia-has-been-confirmed/

^3^ https://www.worldometers.info/coronavirus/country/serbia

^4^ https://www.worldometers.info/coronavirus/

^5^ https://www.gisaid.org

^6^ https://github.com/federicogiorgi/giorgilab/tree/master/coronannotator

^7^ https://doi.org/10.55876/gis8.230901st
