# Supplementary material for: Genome sequence diversity of SARS-CoV-2 in Serbia: insights gained from a 3-year pandemic study
Source: Front Microbiol. 2024 Feb 27;15:1332276. doi: 10.3389/fmicb.2024.1332276 (PMC10929721; doi:10.3389/fmicb.2024.1332276)
Supplement: Supplementary file 1 [file Data_Sheet_1.docx]

Supplementary Material

# Detail for the NGS library preparation and sequencing using three different NGS technologies

**Oxford NANOPORE Technology**

For this study 574 (23.9%) SARS-CoV-2 isolates were sequenced using MinION (Oxford Nanopore Technologies) as described in Vidanovic et al. (2021). This technology was used at the Veterinary Specialized Institute “Kraljevo” (148 samples, 6.2%) and the Institute of Microbiology and Immunology, Department of Virology, Faculty of Medicine, University of Belgrade (426 samples, 17.8%)

Total nucleic acids were extracted using BIOEXTRACT ® SUPERBALL® kit (Biosellal, France) and the Kingfisher Flex device (Thermo Fisher Scientific, Finland), according to the manufacturer’s instructions and SARS-CoV2 was quantified by Real-Time Fluorescent RT-PCR Kit for Detecting SARS-CoV-2 (BGI, China) on the AriaMX Real Time PCR device (Agilent, USA), according to the manufacturer’s instructions.

Synthesis of cDNA was performed using SuperScript IV Reverse Transcriptase (Thermo Fisher Scientific, Finland) and quantified via qRT-PCR assay designed by Pasteur Institute, France, targeting RNA dependent RNA polymerase gene using Luna® Universal Probe qPCR Master Mix (NEB, USA), according to the manufacturer’s instructions.

cDNA was amplified using multiplex ARTIC protocol scheme for SARS-CoV-2 (Version 3) and needed components from the New England Biolabs (USA), then pooled, cleaned up with 1:1 AmpliClean™ Cleanup kit, magnetic beads (Nimagen, USA), and quantified by Qubit Fluorometer using High sensitivity dsDNA assay (Thermo Fisher Scientific, Finland). NGS library was prepared based on the ARTIC protocol using Ligation Sequencing Kit (SQK-LSK109, Oxford Nanopore Technologies) and Native Barcoding Expansion 1-12 (PCR-free) (EXP-NBD104, Oxford Nanopore Technologies) with 7 ng of library loaded on the R9.4.1 flow cell (Oxford Nanopore Technologies), and sequenced on the Oxford Nanopore MinION (Oxford Nanopore Technologies) for 48h.

**Ion Torrent Technology**

For this study 176 (7.3%) SARS-CoV-2 isolates were sequenced using Ion AmpliSeq™ SARS-CoV-2 Research Panel (Thermo Fisher Scientific, Waltham, MA, USA). This technology was used at the Center for Forensic and Applied Molecular Genetics (Faculty of Biology, University of Belgrade).

The viral RNA copy number in samples was determined on the ABI 7500 Real-Time PCR System (Applied Biosystems, USA) using the TaqMan™ 2019-nCoV Assay and Control Kit v1 and the TaqPath™ 1-Step RT-qPCR Master Mix, CG (Thermo Fisher Scientific, Waltham, MA, USA) according to the manufacturer’s recommendations.

After performing reverse transcription with the SuperScript™ VILO™ cDNA Synthesis Kit (Thermo Fisher Scientific, Waltham, MA, USA) and the ProFlex PCR system (Applied Biosystems, USA), we proceeded to the construction of libraries, which consisted of eight samples each.

Library preparation was carried out on the Ion Chef™ instrument (Thermo Fisher Scientific, Waltham, MA, USA) using the Precision ID DL8 Kit (Thermo Fisher Scientific, Waltham, MA, USA) and the Ion AmpliSeq™ SARS-CoV-2 Research Panel (Thermo Fisher Scientific, Waltham, MA, USA).

Barcoded libraries were quantified on the Real-Time PCR System using the Ion Library TaqMan™ Quantitation Kit (Thermo Fisher Scientific, Waltham, MA, USA), diluted to 30pM and pooled in pairs in equal volume aliquots. Templating and sequencing of the Ion 530™ Chips was carried out with the Ion S5™ Precision ID Chef & Sequencing Kit ™ on the Ion Chef™ and the Ion GeneStudio S5™ sequencing system (Thermo Fisher Scientific, Waltham, MA, USA).

**MGI-DNBSEQ Technology**

For this study 1648 (68.7%) SARS-CoV-2 isolates were sequenced using MGI-DNBSEQ technology. For SARS-CoV-2 RNA isolation MGIEasy Nucleic Acid Extraction Kit (MGI Tech Co., Shenzhen, China) was used according to the manufacturer’s instructions. Extractions were performed on MGISP-960 instrument (MGI Tech Co., China). This technology was used at the Center for Genome Sequencing and Bioinformatics at the Institute of Molecular Genetics and Genetic Engineering, University of Belgrade.

cDNA synthesis and its amplification were performed using ATOPlex RNA Universal Library Preparation Module (MGI Tech, Shenzhen, China) and the barcodes were added to the multiplex PCR products using ATOPlex Dual Barcode Primer Module (MGI Tech, Shenzhen, China), all according to the manufacturer’s instructions. Second PCR was purified using ATOPlex RNA Universal Library Preparation Module, samples were quantified on Qubit® 3.0 Fluorimeter using Qubit dsDNA HS Assay Kit (ThermoFisher Scientific, MA, USA) and pooled to reach 400 ng in the total volume of 48 μL. For single-strand circle DNA making MGIEasy Dual Barcode Circularization Module was used according to the manufacturer’s instructions. Preparation of DNA NanoBall (DNB) was performed using DNBSEQ-G400RS High-Throughput Sequencing Kit. To ensure balanced GC content, sample DNB was mixed with Balanced DNB in 3:1 ratio. Afterwards, 30 μL of pooled DNBs was loaded on DNBSEQ-G400RS Sequencing Flow Cell (MGI Tech, Shenzhen, China) and sequenced on DNBSEQ-G400 instrument (MGI Tech, Shenzhen, China) for 45h.

# Supplementary tables

**Supplementary Table 1.** Distribution of SARS-CoV-2 sequences detected in the territory of the Republic of Serbia per clades as defined by Nextstrain and the periods of their detection during three years long study.

| Clade | Number of sequences in total sample of 2396 assigned sequences | Number of sequences in 2107 sequences subset | Detected from date | Detected until date |
| --- | --- | --- | --- | --- |
| 19B | 3 | 0 | April 4th 2020 | May 18th 2020 |
| 20A | 75 | 65 | March 9th 2020 | March 8th 2021 |
| 20B | 165 | 133 | March 9th 2020 | May 2nd 2021 |
| 20C | 1 | 1 | April 14th 2020 | / |
| 20D | 153 | 132 | April 3rd 2020 | February 9th 2021 |
| 20E | 13 | 12 | October 2nd 2020 | February 17th 2021 |
| 20G | 1 | 1 | December 30th 2020 | / |
| 20I | 120 | 119 | December 13th 2020 | August 6th 2021 |
| 21J | 175 | 156 | June 26th 2021 | February 4th 2022 |
| 21K | 127 | 84 | December 12th 2021 | June 18th 2022 |
| 21L | 499 | 497 | January 30th 2022 | The end of study |
| 22A | 23 | 22 | May 13th 2022 | September 28th 2022 |
| 22B | 889 | 825 | June 2nd 2022 | The end of study |
| 22C | 26 | 26 | May 22nd 2022 | July 17th 2022 |
| 22D | 21 | 6 | October 14th 2022 | The end of study |
| 22E | 62 | 14 | August 22nd 2022 | The end of study |
| 22F | 30 | 6 | November 18th 2022 | The end of study |
| recombinants | 13 | 8 | April 1st 2022 | The end of study |
